# Toward a Systematic Approach to Generating Demand for Voluntary Medical Male Circumcision: Insights and Results From Field Studies

**DOI:** 10.9745/GHSP-D-15-00020

**Published:** 2015-06-12

**Authors:** Sema K Sgaier, James Baer, Daniel C Rutz, Emmanuel Njeuhmeli, Kim Seifert-Ahanda, Paulin Basinga, Rosie Parkyn, Catharine Laube

**Affiliations:** a Bill & Melinda Gates Foundation, Global Development Program, Integrated Delivery, Seattle, WA, USA, and University of Washington, Department of Global Health, Seattle, WA, USA. Now with Surgo Foundation, Seattle, WA, USA, and Harvard T.H. Chan School of Public Health, Boston, MA, USA; b Bill & Melinda Gates Foundation, Independent Consultant, London, UK; c US Centers for Disease Control and Prevention, Atlanta, GA, USA; d US Agency for International Development, Division of Global HIV/AIDS, Washington, DC, USA; e Bill & Melinda Gates Foundation, Global Development Program, Integrated Delivery, Seattle, WA, USA; f BBC Media Action, London, UK; g US Department of State, Office of the US Global AIDS Coordinator, Washington, DC, USA

## Abstract

By the end of 2014, an estimated 8.5 million men had undergone voluntary medical male circumcision (VMMC) for HIV prevention in 14 priority countries in eastern and southern Africa, representing more than 40% of the global target. However, demand, especially among men most at risk for HIV infection, remains a barrier to realizing the program's full scale and potential impact. We analyzed current demand generation interventions for VMMC by reviewing the available literature and reporting on field visits to programs in 7 priority countries. We present our findings and recommendations using a framework with 4 components: insight development; intervention design; implementation and coordination to achieve scale; and measurement, learning, and evaluation. Most program strategies lacked comprehensive insight development; formative research usually comprised general acceptability studies. Demand generation interventions varied across the countries, from advocacy with community leaders and community mobilization to use of interpersonal communication, mid- and mass media, and new technologies. Some shortcomings in intervention design included using general instead of tailored messaging, focusing solely on the HIV preventive benefits of VMMC, and rolling out individual interventions to address specific barriers rather than a holistic package. Interventions have often been scaled-up without first being evaluated for effectiveness and cost-effectiveness. We recommend national programs create coordinated demand generation interventions, based on insights from multiple disciplines, tailored to the needs and aspirations of defined subsets of the target population, rather than focused exclusively on HIV prevention goals. Programs should implement a comprehensive intervention package with multiple messages and channels, strengthened through continuous monitoring. These insights may be broadly applicable to other programs where voluntary behavior change is essential to achieving public health benefits.

## Abstract (precis)

Using an analytical framework to design and implement voluntary medical male circumcision (VMMC) programs can lead to more effective interventions, especially when insights are incorporated from disciplines such as behavioral science and commercial market research. Promising VMMC behavior change practices: (1) address individual, interpersonal, and environmental barriers and facilitators; (2) tailor messages to men’s behavior change stage and focus on other benefits besides HIV prevention, such as hygiene and sexual pleasure; (3) include women as a key target audience; (4) engage traditional and religious leaders; (5) use media to promote positive social norms; and (6) deploy community mobilizers to address individual concerns.

## INTRODUCTION

Generating demand for voluntary medical male circumcision (VMMC) is a key component of HIV prevention in 14 priority countries with high HIV prevalence in eastern and southern Africa.^1^^,^^2^ Modeling studies conducted in 2009–2011 established that if 80% of males aged 15 to 49 years in these priority countries were circumcised within 5 years, and if coverage levels were maintained thereafter, 3.4 million HIV infections could be prevented over 15 years, saving lives as well as US$16.5 billion in HIV treatment costs.^3^ Global public health leaders have endorsed an overall goal of achieving 20.3 million male circumcisions between 2012 and 2016.^2^

If 80% of men in 14 priority African countries were circumcised, 3.4 million HIV infections could be prevented over 15 years.

VMMC scale-up has been variable among the 14 priority countries, but overall momentum has been building since early 2013. The World Health Organization provisionally estimates that 8.5 million circumcisions had been completed by the end of 2014—an increase of 2.5 million over 2013.^4^ VMMC scale-up is close to reaching half of the overall goal and will have tangible public health benefits.

A wide range of demand generation activities has contributed to the progress of VMMC scale-up.^5^ However, most of the 14 countries are running behind the pace needed to reach their VMMC goals. Apart from adequate funding and service delivery capacity, a substantial increase in demand for VMMC is also essential.

Most of the 14 priority countries are running behind the pace needed to reach their VMMC goals.

The VMMC demand generation effort faces unique challenges. First, research suggests that men are less likely than women to seek health care.^6^ Second, VMMC is a “hard sell” because it requires healthy men to undergo a surgical procedure involving appreciable discomfort and inconvenience and which offers only partial protection against an uncertain (and often unacknowledged) individual HIV risk.^2^ Third, while circumcision is a centuries-old practice with established cultural and religious significance and sensitivity, its ability to reduce risk of HIV and other sexually transmitted infections (STIs) for males has only recently become widely recognized. Scaling-up VMMC, therefore, requires reorienting long-held beliefs to include an appreciation of its protective health benefits. Finally, experience has shown that current approaches to demand generation are often inconsistent, not evidence-based, and poorly coordinated. Political and social factors, including ignorance of the need for strategic demand generation, may contribute to inadequate funding and focus.

There is growing consensus among stakeholders that more strategic use of demand generation resources and opportunities is needed, taking into consideration fresh perspectives and disciplines, to improve uptake of national VMMC programs.^7^ In this paper, we review the available literature on VMMC demand generation and report on field visits to VMMC demand generation programs in 7 countries to assess current strategies and identify gaps, and we suggest fresh approaches to better motivate VMMC candidates to accept services. We do not attempt a systematic review of current VMMC demand generation approaches because there is not yet an extensive or strong evidence base for such a review. Instead, we leverage our extensive collective experience as funders and implementers in the field to present some promising practices and situate these within a framework that may be useful for the systematic creation of more effective and efficient demand generation strategies. The approach and lessons from VMMC scale-up may also be applicable to other public health programs seeking new or improved evidence-based approaches to increase service uptake, retention, and adherence.

## FRAMEWORK FOR VMMC DEMAND GENERATION

**FIGURE 1.**
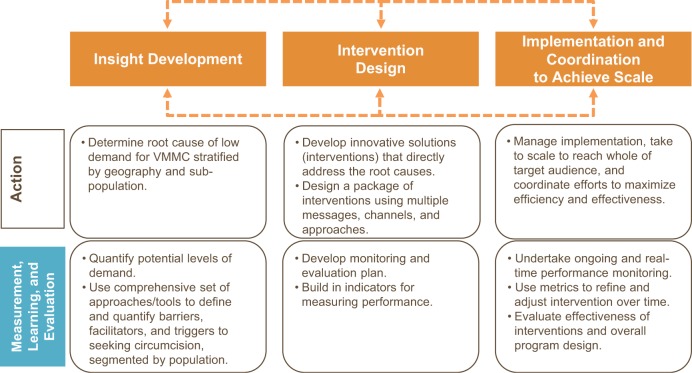
Components of Demand Generation for Voluntary Medical Male Circumcision (VMMC)

In this paper, we present our findings and insights in terms of a framework for VMMC demand generation that draws from elements of other behavior change theories and frameworks. Our illustrative VMMC demand generation framework has 4 components: (1) insight development, (2) intervention design, (3) implementation and coordination to achieve scale, and (4) measurement, learning, and evaluation (Figure 1). The first 3 components are interdependent and may overlap. The fourth component underpins the other components, since measurement, learning, and evaluation are foundational for all stages of demand generation.

**Insight development.** This foundational phase of VMMC demand generation programs consists of a process of conducting quantitative and qualitative research (i.e., formative research) to understand what drives or limits demand for VMMC and how VMMC can be framed for greatest appeal at both the individual and the population levels. Established theories of behavior change provide a roadmap for how VMMC candidates may journey toward the commitment to seek services—progressing from unawareness to awareness (sometimes coupled with apathy or opposition), followed by interest, preparation, and finally action.^8^^–^^11^

**Intervention design.** Innovative solutions to generate demand do not focus simply on the public health benefits of VMMC, but address the cognitive, emotional, cultural, and structural barriers that can hinder a man’s decision to be circumcised—and the corresponding triggers that can facilitate that decision. Successful interventions tend to use carefully designed and coordinated messages delivered via multiple channels.

**Implementation and coordination to achieve scale.** Most effective demand generation activities must be delivered at scale, combining high levels of coverage and intensity. This requires more than money: it involves sound implementation management to ensure coordination among key VMMC stakeholders.

**Measurement, learning, and evaluation.** The environment in which public health programs operate and their beneficiaries live is complex and evolving, and demand for VMMC changes accordingly. A robust, dynamic approach is needed to gather data on levels of demand and on the effectiveness and cost-effectiveness of demand generation programs. Given that global and country-level scale-up targets were originally developed without taking into account the interaction between demand- and supply-side interventions, key questions for VMMC programs are: How much demand is realistic in the context of available supply and the known level of interest at a given time, and how should this level of demand be defined and measured? Setting a demand-based denominator helps establish feasible targets and provides metrics to measure the effectiveness of specific demand generation activities.

## METHODS

We reviewed available literature on VMMC demand generation programs, including peer-reviewed publications, gray literature, and a 2013 landscape analysis and documentation.^1^^,^^12^^–^^15^ We also conducted field studies of VMMC demand generation programs over a 4-month period during 2013–2014 in 7 of the priority countries for VMMC^2^—Kenya, Malawi, South Africa, Tanzania, Uganda, Zambia, and Zimbabwe.^16^

For the field studies, following desk research, we mapped current demand generation practices for VMMC in each of the 7 countries. We then reviewed 45 interventions and held 112 in-country interviews with implementers and other stakeholders to identify examples of promising practices for wider learning and potential scale-up (see supplementary materials for the phase 1 discussion guide). A brief overview of each example was presented at a meeting of researchers, implementing partners, and government and donor representatives, held in Lusaka, Zambia, in April 2014.^7^ The research team then returned to each country to document each promising practice in greater detail (see supplementary materials for the phase 2 discussion guide).

## FINDINGS

The literature review produced scant evidence on approaches to demand generation for VMMC, both in terms of understanding drivers of demand and in terms of evaluating existing interventions. Based on our field studies, we produced 27 implementation profiles of interventions that were sufficiently developed to inform adoption, adaptation, and scale-up by colleagues involved in VMMC demand generation in eastern and southern Africa.^16^ The Table provides a summary of the 27 interventions. The findings and recommendations we present here are based primarily on the 27 implementation profiles documented through the field studies and on the gaps revealed by the literature review, as well as on our own field experience.

**TABLE.** Summary of 27 Promising-Practice Interventions for VMMC

**Country and Name/Description of Intervention**	**Types of Interventions**	**Implementing Partner**	**Brief Description**
**KENYA**			
Demand creation toolkit	Research, messaging, social mobilization, IPC	Impact Research and Development Organization (IRDO)	Tool to assist social mobilizers to communicate consistent messages during IPC
Journalism training	Media	Internews	Training journalists to report accurately and impartially on VMMC
MCC coordination	Coordination	National AIDS & STI Control Programme (NASCOP)	National and provincial coordination
**MALAWI**			
BRIDGE II project	Social mobilization, IPC, engaging traditional leaders and female partners, coordination, messaging incentives	Johns Hopkins Center for Communication Programs (CCP)	Combining low- and high-intensity efforts at behavior change, balancing supply and demand, and reaching female partners
Lilongwe district scale-up	Social mobilization, media, IPC, engaging traditional leaders and female partners, coordination	International Training & Education Center for Health (I-TECH)	Using satisfied VMMC clients as community mobilizers and training women to reach other women
**SOUTH AFRICA**			
Brothers for Life	Social mobilization, IPC, media, ICT, messaging	Johns Hopkins Health and Education in South Africa (JHHESA)	Award-winning multipronged and adaptable marketing and awareness campaign involving print media, TV, billboards, community outreach projects, canvassing, education, and information
CareWorks	Social mobilization, IPC, ICT, incentives payment	CareWorks	Workplace programs and a call center to move potential clients from contemplation to action
Centre for HIV and AIDS Prevention Studies (CHAPS)	Social mobilization, IPC, research, incentives and performance-based payment	CHAPS	Record numbers of VMMCs performed, perpetual research, reflection and revision of strategy, tight feedback loop with mobilizers, collective incentive structure translated into highly motivated staff
New Start	Social mobilization, engaging traditional leaders, messaging, research, media	Society for Family Health (SFH)	Use of the DELTA process to design demand creation interventions and pretesting of all materials
Soul City	Research, messaging, media	Soul City	Insertion of VMMC storylines into long-running TV series
**TANZANIA**			
Champion project	Social mobilization, IPC, engaging female partners, media	EngenderHealth	Beyond HIV messaging, engendering long-term commitment of social mobilizers
Community mobilization and GIS technology	Social mobilization, IPC, media, engaging female partners, research, advocacy, IPC	Jhpiego	Community mobilization for older men, use of SMS and GIS technology
Community mobilization and SMS technology	Social mobilization, IPC, ICT	IntraHealth and Tanzania Youth Alliance (TAYOA)	Innovative approach combining SMS technology with popular-opinion leaders at grassroots level to create tailored strategy for reaching older men, adaptation of IEC materials to suit local communities
Printed materials and radio spots	Media and messaging	CCP	Printed materials and radio spots
**UGANDA**			
AMAKA (Adult Male Medical Circumcision in Kampala)	Social mobilization, research	Infectious Diseases Institute	Use of existing community resources and structures for demand creation
Be the Pride of Your Tribe campaign	Social mobilization, advocacy, research	STAR-E	VMMC campaign in traditionally circumcising communities
Makerere University Walter Reed Project	Social mobilization, advocacy, media, IPC, research	US Military HIV Research Programme	Use of cross-country learning to create VMMC demand and meet VMMC need
Stand Proud, Get Circumcised campaign	Research, messaging, social mobilization	Health Communication Partnership (HCP)	Use of research to inform communication strategy development and implementation, national tools with standardized branding and information to ensure consistency and recognition of VMMC but which can also be tailored to specific communities and contexts
Stylish Man campaign	Research, media, messaging, IPC	Rakai Health Sciences Program	Demedicalizing demand creation for VMMC
**ZAMBIA**			
Community mobilization	Social mobilization, IPC, media, engaging female partners	Marie Stopes International	Community mobilization, health counselor training, and local media
Community mobilization and campaigns	Social mobilization, research, ICT	Society for Family Health	Community mobilization through existing volunteer structures, mobile clinical outreach, client voucher/feedback system to disaggregate effective elements of demand creation, and toll-free family planning help lines incorporate VMMC
Mini campaigns and training	Social mobilization. IPC, media, advocacy	Jhpiego	Close linking of demand creation activities with supply, including mini campaigns to marry the two and emphasis on a positive client experience
National MC Month and technical working group	Mass media communication, advocacy, engaging tribal leaders, community mobilization, IPC	National technical working group	National and district-level coordination, advocacy by traditional leaders and public officials, thrice yearly MC drives and engagement with health workers
Safe Love campaign	Research, media, messaging, IPC, ICT, community mobilization	Communications Support for Health	Innovative multiplatform, multi-format content, use of SMS to move from contemplation to action
U Report SMS pilot	ICT, advocacy, community mobilization, IPC	United Nations Children’s Fund (UNICEF)	SMS pilot for VMMC demand creation among adolescents and young adults
**ZIMBABWE**			
Identifying optimal messaging	Research, messaging	Battelle Health and Analytics	Qualitative and quantitative research to establish optimal messages to promote demand for VMMC
SMART campaign	Messaging, media, social mobilization, IPC	Population Services International (PSI)	Research-led message development for “SMART” campaign, management of social mobilization

Abbreviations: GIS, geographic information system; ICT, information and communications technology; IEC, information, education, and communication; IPC, interpersonal communication; MC, male circumcision; SMS, short message service (text message); VMMC, voluntary medical male circumcision.

### Insight Development

Our analysis and experience suggest that the extent to which programs currently apply behavior change theories to their VMMC demand generation efforts varies, due to limited expertise, constraints on time, budget, or feasibility, or a combination of these factors. While some degree of research is conducted in most settings, many programs lack a sufficiently systematic and granular view of how men progress through the different stages of behavior change and of all the influences along this path.

The formative research that has been conducted has consisted primarily of acceptability studies in several of the VMMC priority countries, focusing on the perceived barriers and benefits to VMMC.^15^ These studies have favored qualitative methods, using in-depth interviews, focus groups, or self-administered surveys.^17^ Acceptability of VMMC has been found to vary by demography and geography. In general, studies have found acceptability to be higher among females and males aged 15–25 years than among older people,^18^^,^^19^ and to be higher in eastern Africa than in southern Africa.^20^^,^^21^ Commonly identified factors that make VMMC acceptable to males included supportive social norms demonstrated by peers, family, or female partners. Commonly cited barriers included cultural and traditional norms, fear of pain, length of the healing period, perceived threat to masculinity, and financial and other opportunity costs.^15^ These triggers and barriers varied by age: adolescents were likely to be more amenable to peer pressure or parental persuasion than older men, while older men were more likely than adolescents to view financial costs and sexual abstinence during the recovery period as barriers.

VMMC acceptability is generally higher among younger than older men and women.

**FIGURE 2.**
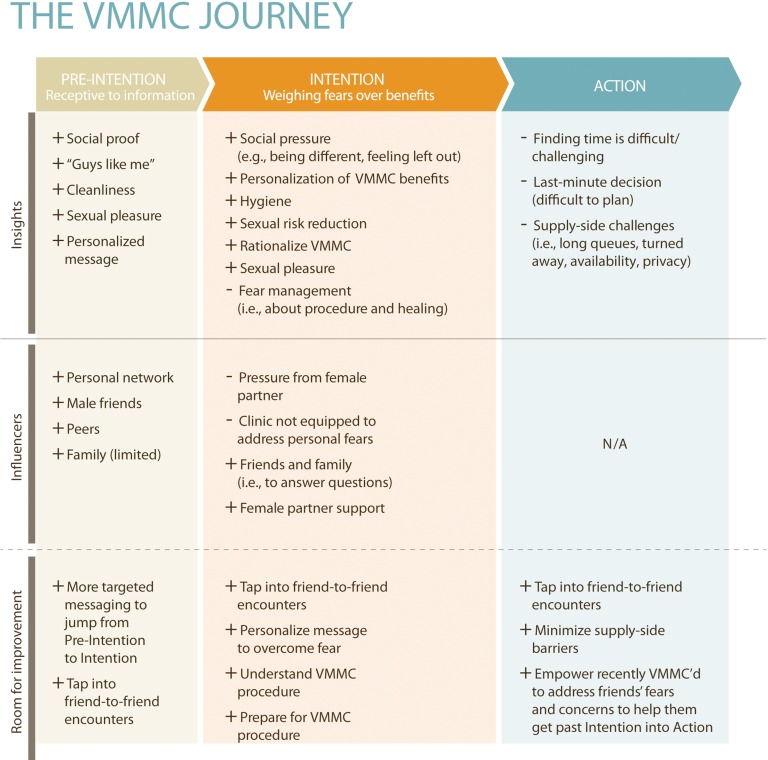
Behavior Change Continuum for Voluntary Medical Male Circumcision (VMMC), Based on Insights from VMMC Program in Zimbabwe Key: +, motivators; –, barriers.

A limitation of acceptability studies is that the insights they provide are general, and the studies are thus often only useful for designing interventions that move an individual from opposition or apathy toward VMMC to a state of interest in the procedure. A few recent studies in Zimbabwe have described the triggers that influence males to seek VMMC once they are interested in the procedure and understand its benefits.^11^^,^^13^^,^^22^ These studies have identified a dominant pattern in which behavior change occurs along a continuum of 3 fluid stages—pre-intention, intention, and action (Figure 2).

One of the studies identified interventions that are likely to move men more quickly through this process of change.^23^ It emphasized the value of tailoring messages and activities to the men’s stage-specific needs and concerns. For example, during the pre-intention stage, the aim of behavior change interventions should be to increase men’s exposure to VMMC messages through multiple channels, then make VMMC relevant to individuals through targeted messaging. Social pressure and encouragement to ascribe positive values to VMMC help move men further toward the decision to act, and addressing men’s fears about the procedure may remove the final barrier. Male friends who have undergone circumcision and female partners are the key influencers throughout the process. At the same time, more should be done to provide information about the procedure itself and to address the supply-side issues that prevent many males interested in VMMC from accessing it.

Messages should be tailored to men’s behavior change stage.

Overall, most formative research studies fall short in two ways. First, there is growing acknowledgment that psychographic variables such as attitudes, perceptions, aspirations, emotions, biases, and mental models play a very important role in decision making: most decisions are not rational.^24^ However, the studies we reviewed were not designed to uncover such variables. Second, given the heterogeneity of VMMC’s target population and the people and institutions that may influence these populations, generalized results from acceptability studies are likely insufficient for designing strategies to reach members of a specific sub-population with compelling information that meets them “where they are.” It is this failure to understand the precise needs and concerns of segmented sub-populations of males, and to design interventions and messaging accordingly, that likely accounts for the significant gap between interest in VMMC and actual uptake of the procedure.

### Intervention Design

Our analysis identified different demand generation interventions used by VMMC priority countries to address the barriers and facilitators to VMMC identified in acceptability studies. The interventions included^5^:

- Advocacy with community leaders (political, traditional, or religious) and through existing community structures
- Community mobilization, including engagement of women
- Interpersonal communication (IPC) through community mobilizers
- Mid- and mass media campaigns
- Use of information and communications (ICT) technologies

Many interventions combined different techniques and approaches, which were likely to be more effective the more closely they were aligned with one another. However, most interventions have not yet been formally evaluated. Box 1 provides a summary of the key promising practices.

**BOX 1. Promising Practices for Voluntary Medical Male Circumcision (VMMC) Demand Generation**

- **Tailoring messages carefully to the audience.** Find the most appropriate message for the demographic—and for the individual’s place on the continuum of pre-intention, intention, and action behavior change stages.
- **Reaching out to traditional leaders.** Tribal and religious leaders can change an existing norm, give reassurance, and lend authority to a program.
- **Using mobilizers.** Interpersonal communication can address the client’s individual concerns.
- **Targeting women as an audience for messaging and using them as change agents.** Women (wives, girlfriends, and mothers) can be change agents; the protective benefits of VMMC are important to them, too.
- **Appealing to reasons/motivations other than HIV prevention.** Men’s motivations to get circumcised may be other than protection for HIV; more near-term benefits such as protection from STIs might be more relevant, and VMMC may be seen as “modern” or aspirational. The appeal of belonging to a group can also be persuasive.
- **Packaging VMMC with other interventions.** Men undergoing HIV testing and counseling, or women receiving treatment for prevention of mother-to-child transmission of HIV, may be open to a VMMC referral.
- **Using new technologies.** Messaging delivered via the Internet, social media, and mobile phones should be part of the mix along with traditional media.

Many countries have found that **engaging with traditional leaders** can provide reassurance to VMMC candidates that being circumcised will not change their ethnic, religious, or cultural identity. In Malawi, early and ongoing engagement with leaders of traditionally non-circumcising communities has been instrumental in mobilizing community-level support for the VMMC program and in encouraging males to undergo circumcision.^25^ In eastern Uganda, safe male circumcision was promoted among traditionally circumcising communities through the “Be the Pride of Your Tribe” campaign, which engaged religious and tribal leaders as well as health care providers.^26^

Early engagement with leaders of traditionally non-circumcising communities in Malawi has been key to mobilizing VMMC support.

The deployment of **community mobilizers** to undertake one-on-one messaging with prospective clients has been one of the most consistently employed and effective strategies across the region.^5^ This kind of IPC allows men who may be contemplating the procedure to ask questions specific to their own concerns in privacy. It works best when mobilizers use a standardized, comprehensive discussion guide and package of information.^27^ In South Africa, training, strong management oversight, and a collective incentive structure strengthened the effectiveness of community mobilizers.^28^ In Malawi, satisfied clients have been employed as mobilizers.^29^ In Kenya, the Impact Research and Development Organization (IRDO) produced an IPC toolkit to support counselors in conveying information tailored to the individual’s stage in the decision-making process.^30^ In Zambia, Society for Family Health (SFH) has used the community engagement methodology of education through listening, which emphasizes the need to draw out and address individual barriers.^31^ Counseling women on the benefits of VMMC is recognized as an important facilitator of VMMC uptake in most of the priority countries, and the Kenya program has gone a step further in using married women to educate other women and couples about VMMC in women’s groups, antenatal clinics, and other health care settings.^32^

Deployment of community mobilizers to address men’s individual concerns has been one of the most effective VMMC strategies.

**Media** work has included training Kenyan journalists to report accurately on the science behind VMMC^33^; use of radio magazine broadcasts in Zambia incorporating key messaging on VMMC^34^; and radio spots and print materials tailored to different regions of Tanzania, featuring the voices of “satisfied customers” and local health experts.^35^ Generally, implementers working through the media have sought more resources to roll out campaigns and to achieve greater coverage. In Malawi, campaigns were ramped up to correspond to periods of increased supply^29^; elsewhere, mobile vans have been used to meet increasing demand.^36^ In Tanzania, a program uses a geographic information system (GIS) to map its VMMC activities, including the location of facilities and infrastructure, to help determine where to conduct demand generation activities and to identify unreached populations.^37^

These examples show that implementers have acted on several behavioral determinants to drive demand for VMMC, but the analysis of these determinants in the planning stage has not always been rigorous. Ideally, interventions should incorporate formative research during the design phase to understand the cognitive and social barriers and motivators to undertaking VMMC. In Zimbabwe, for example, Population Services International (PSI) conducted such research through in-depth interviews as opposed to focus groups, arguing that interviews deliver a deeper, more truthful insight into these barriers and motivators than focus groups.^13^^,^^27^ PSI also conducted a TRaC (Tracking Results Continuously, a multi-round survey-based research approach) study in Zimbabwe, which segmented respondents according to their stage in the decision-making process.^13^^,^^27^ Media outputs should be pretested, and interventions should be continually evaluated once implementation is underway to enable course correction and to develop a clear evidence base to support scale-up.

There are other ways in which intervention design has fallen short. First, many country programs have attempted to reach all audiences with a standard set of promotional materials and to apply general messaging in all demand generation activities. While it is vital to try to achieve scale at the national level, this “one-size-fits-all” approach fails to recognize the difference in attitudes, knowledge, and favored communication channels of, for example, a rural Kenyan schoolboy compared with a professional man in the capital city of Nairobi. It is entirely possible to reach large numbers of people with a single brand identity while targeting and tailoring communications to specific groups within that brand.

National branding of VMMC should be combined with tailored messaging for different groups of males.

Second, most demand generation messaging has focused on the programmatic and public health imperatives of VMMC, emphasizing prevention of HIV and other STIs,^38^ rather than the values, perceived needs, and aspirations of men. While many VMMC clients may understand the protective benefits of circumcision (which should continue to be explained in counseling sessions before the procedure), they are more likely to consider VMMC for other reasons, including hygiene, pleasing a sexual partner, and conforming to peer norms.^39^ The Rakai Health Sciences Program has been conducting safe male circumcision in Uganda since 2003 but experienced a demand plateau in 2013. Research suggested that the protective benefits were well understood by men and that future campaigns could overcome that plateau by presenting VMMC as an aspirational procedure.^40^

Many men are more likely to consider VMMC for other reasons besides HIV prevention.

Third, programs have often been rolled out individually to address specific barriers to demand, rather than forming part of a package of interventions that holistically address all the touch-points along the behavior change continuum for VMMC. PSI’s work in Zimbabwe is a good example of how IPC can be deployed as an effective supplement to mass media interventions: the media element supports development of supportive social norms and sparks private conversations with social mobilizers, religious and community leaders, partners, and peers that could result in a decision to undertake VMMC.^27^ In South Africa, the Centre for HIV and AIDS Prevention Studies (CHAPS) regards the quality of the service itself as a crucial touch-point. To illustrate, a client who attends a VMMC clinic has already made the decision to undertake VMMC, but his experience at the clinic will inform whether he recommends the procedure to his peers.^41^

Finally, many programs would benefit from paying more attention to the value of discussion, as opposed to direct messaging, in driving the development of supportive social norms around VMMC. Discussion between prospective clients and counselors or peers is already proving effective in addressing individual concerns and questions a client may have about the procedure, but the media may also have a role to play in sparking or providing a space for audiences to engage in public discussion about VMMC.^42^ For example, in India, where people were embarrassed to say the word condom, a condom normalization campaign humorously encouraged people to shout it in public.^43^

### Implementation and Coordination to Achieve Scale

As with most health interventions, plans for VMMC scale-up generally have discounted demand-side challenges.^2^^,^^44^ Regional and national VMMC strategies have set targets based on epidemiologic considerations, rather than on demand forecasts or other feasibility considerations, even where these constraints are acknowledged.^2^ Few national VMMC operational plans have developed comprehensive demand-side strategies based on current data.^45^^,^^46^ There is insufficient understanding of the proportion of program funds needed for demand generation and how this may vary for easy- versus hard-to-reach populations.^47^ Demand generation is frequently perceived as expensive, even though evidence from VMMC and other public health programs suggests that up-front investment in demand generation activities gradually reduces the cost per service provided by fueling higher sustained volume and thus greater efficiency.^48^^,^^49^ In fact, clinical service volume creates more variability in VMMC unit costs than any other variable.^50^^,^^51^ But promising interventions have not always been implemented at scale, thereby diminishing their potential impact.^7^

Few national VMMC operational plans have developed comprehensive demand-side strategies.

Demand generation interventions, particularly those employing mass media communications, can reach far beyond a service catchment area, necessitating intensive coordination to ensure activities are mutually reinforced across sites and implementers—that is, that they disseminate consistent and complementary messaging, enable efficient client referrals, and reach but do not oversaturate crucial stakeholders and priority audiences. Ideally, national VMMC operational plans would assign the geographic focus, roles, and responsibilities of demand generation partners and identify standing coordination mechanisms to ensure programs remain aligned as the demand generation strategies evolve. Kenya has such a mechanism in place: the National AIDS & STI Control Programme (NASCOP) convenes quarterly meetings with national and provincial VMMC implementing partners to coordinate their efforts and plan joint initiatives. In addition, a Male Circumcision Consortium provides technical support to NASCOP to enhance coordination and quality assurance.^52^

### Measurement, Learning, and Evaluation

Evaluation of VMMC demand generation programs is essential, but few interventions have been evaluated for effectiveness, and fewer still for cost-effectiveness. In Zimbabwe, a cluster randomized controlled trial (RCT) tested the effectiveness of the “Male Circumcision Uptake Through Soccer” program, in which soccer teams were randomized to a brief VMMC education and motivation intervention.^53^ Those receiving the intervention were relatively more likely to take up VMMC, although the absolute effect was small. In Kenya, an RCT found that conditional economic compensation (in the form of food vouchers) to address reported economic barriers to VMMC uptake led to a modest increase in circumcision coverage among men aged 25–49 years.^54^ Five other interventions that explore the use of incentives, advertising, and ICT platforms have been evaluated (publication of results is pending).^55^ Nevertheless, there is a need for systematic evaluation to be consistently included in demand generation programs, including evaluating a package of interventions for their collective effectiveness.

Equally important is the ability to monitor the performance and cost of demand generation interventions as they are being introduced and scaled-up, using key performance indicators to assess strategies and inform the program throughout its course. Typically, programs have judged the performance of demand generation interventions by assessing VMMC outputs (i.e., the number of men circumcised). However, this is an unreliable approach since multiple demand- and supply-side variables contribute to these results, making it difficult to judge the performance of a specific factor. In a study in Zambia, 17 of 40 interviewed men noted they had had 1 to 4 failed prior attempts to get circumcised, suggesting significant supply-side issues creating unmet need.^23^

## DISCUSSION

While relatively few VMMC demand generation programs have been formally evaluated, our research identified key approaches that we believe will make interventions more effective and cost-effective. These approaches are summarized in Box 2 and described in detail below in terms of the framework for VMMC demand generation.

**BOX 2. Recommendations for an Innovative and Systematic Approach to VMMC Demand Generation**

- **Measure demand and set appropriate targets.** Supplement existing research with fresh assessments where needed; ensure that targets take into account supply-side capacities.
- **Place the customer at the center of the campaign.** Segment target audiences by psychographic variables as well as demographic ones, and tailor strategies to each audience.
- **Take a multidisciplinary approach.** Tap into disciplines beyond public health such as behavioral economics, the science of decision making, private-sector market research methods, and network analyses.
- **Design and test interventions.** Use formative research; test effectiveness of interventions; adjust as needed based on data before scaling-up.
- **Apply a comprehensive demand generation strategy.** Use multiple messages, channels, and approaches, coordinated to be mutually reinforcing.
- **Invest in data monitoring systems.** Ensure there is real-time and more proximal feedback on intervention outputs.
- **Strengthen program management.** Analyze the cost of demand generation activities, and advocate increased funding to take effective interventions to scale.
- **Ensure coordination between donors, governments, and implementing partners.** Avoid a patchwork of initiatives: ensure that interventions are consistent and complementary and that roles and responsibilities allow partners to contribute from their core areas of expertise.

### Insight Development: Borrow Behavior Change Theories and Tools From Other Disciplines to Understand Demand More Systematically

**FIGURE 3.**
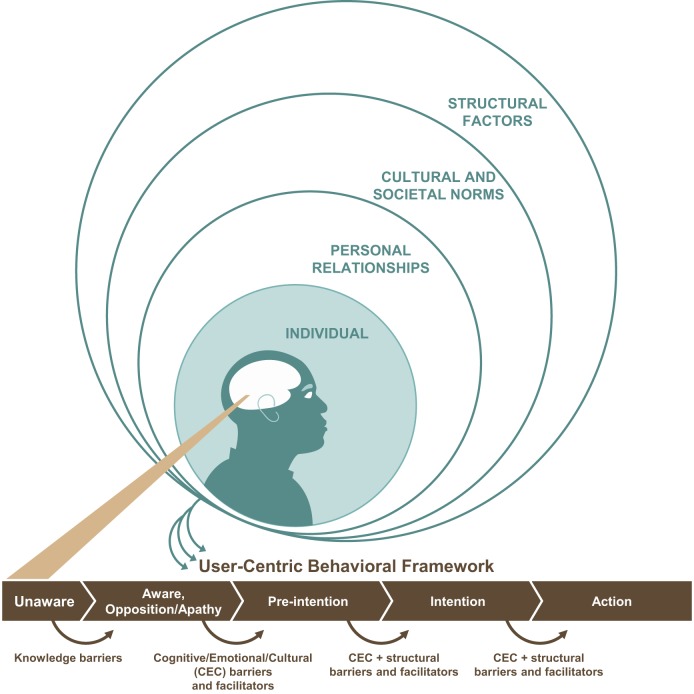
User-Centric Behavioral Framework for Voluntary Medical Male Circumcision

In addition to stage models of behavior change,^8^^–^^11^^,^^56^^,^^57^ a socio-ecological model can provide insight into the individual and interpersonal factors, community and organizational influences, and public policies that shape behaviors.^58^^–^^60^ Some implementing partners use one or more of these models, but blending these into a single behavioral framework can unify, systematize, and strengthen the behavior change strategy. A “user-centric” framework, such as the illustrative example in Figure 3, describes the steps in a typical client’s progression to changed behavior, with a focus on the individual’s perceived VMMC values and needs. At each step, internal factors (e.g., cognitive and emotional) as well as environmental ones (e.g., cultural and ethical factors or issues around service delivery) influence the individual’s immediate needs and wants, which, in turn, govern subsequent actions.

While stage and socio-ecological models of behavior change typically underpin social and behavior change communication, we can open the door to a wider range of novel, effective, and efficient demand generation interventions by borrowing formative research tools from disciplines outside classic public health communication. Specifically, research into decision making and behavioral economics reveals a myriad of factors—biological, cognitive (conscious and subconscious), social, cultural, and economic—that drive decision making.^61^^,^^62^ For example, in rural Rajasthan, India, families were deterred from completing the full immunization schedule for their children by high transportation costs and wage loss. Offering small non-cash incentives (raw lentils and metal plates) proved considerably more effective than simply improving the reliability of services.^63^ In Vietnam, social norms were identified as one of the barriers to initiation of early and exclusive breastfeeding.^64^

Commercial marketing takes a systematic, evidence-driven approach to identifying and fulfilling consumer needs and aspirations.^65^ Key insights, identified through qualitative and quantitative market research, measure and prioritize market opportunities.^66^^–^^68^ This translates to the public health field, where, for example, individual adults’ acceptance of influenza vaccine has been found to be correlated with positive community attitudes, as well as with the individual having previously had a flu shot; this can enable officials to identify potential markets for flu vaccination and target them with appropriate messaging.^69^ Potential customers can be segmented and prioritized according to their values, opinions, attitudes, interests, and lifestyles.^70^ Similarly, market research into demand for VMMC can offer an evidence-based understanding of how men progress through the stages of awareness, interest, preparation, and action (Figure 3).

### Intervention Design: Consider Evidence From Insight Development to Develop Effective Interventions

It is important that programs consider evidence about cognitive, emotional, cultural, and structural barriers to VMMC to develop and fine-tune their intervention strategies. Knowledge gained from the insight development phase can reveal the best platforms and channels for delivering the right message in the right way and at the right time. For example, the word of traditional leaders is extremely powerful in some communities. Effective platforms may also include mass and mid-media, IPC, social media, and other new technologies. IPC needs to be localized and personalized. With a strong emphasis on training, management, and use of data, IPC platforms can be scaled-up effectively, even in large, far-flung programs. Tailored training of community mobilizers and other IPC agents can enable them to better share information about VMMC with diverse population segments.^71^^,^^72^ For example, the Avahan HIV prevention program in India found that illiterate sex workers, when mobilized as peer outreach workers, were able to track the number and content of their meetings with their outreach contacts via tools that used symbols and stickers, when trained to use them in a standardized manner.^73^ We can envisage simple, research-based tools that would enable community mobilizers to identify a man’s precise position along the VMMC behavior change continuum (Figure 3) on the basis of a small number of screening questions. The community mobilizer could then use IPC tools (scripts, questions, discussion points, etc.) to address the barriers relevant to the man at that time and to strengthen triggers for positive action. The experience of Kenya provides an example of how a country has used evidence to inform its demand generation activities, which evolved over the course of the program to address specific subgroups of the population (Box 3).

**BOX 3. Kenya Case Study: Using Evidence to Evolve VMMC Demand Generation Strategies**

The Kenya VMMC program, which has achieved about 70% VMMC coverage, against its target of 80%, provides a good example of how programs should use evidence to inform their demand-side response and to evolve over the course of the program.

**FIGURE 4.**
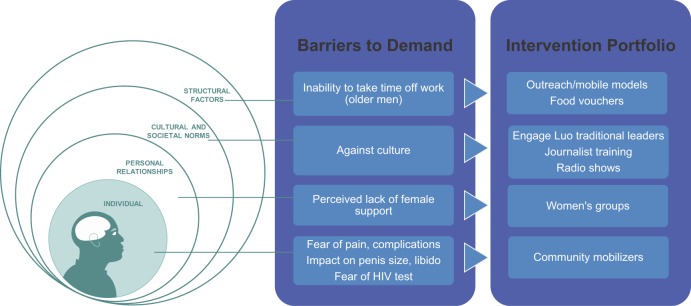
Addressing Barriers to Voluntary Medical Male Circumcision in Kenya: An Evidence-Based, 360°, Well-Coordinated, Evolving Approach

First, formative research identified a number of individual, interpersonal, cultural, and structural barriers to circumcision, and the program developed different interventions to address each of these barriers (Figure 4). One of the main barriers to circumcision was cultural—the Luo tribe was traditionally non-circumcising, differentiating the Luo from other tribes. Demand-side interventions in the early days of the VMMC program focused on working with the traditional leaders of the Luo community in Nyanza to build a strong enabling environment for the program and to address traditional barriers to uptake.

As these community-level barriers were broken, the program then focused on one-to-one promotion to address individual barriers (such as fear of pain) by deploying a massive and incentivized outreach campaign with community mobilizers. Journalist training and radio shows ensured a positive “noise” in the environment.

Currently in its final stage, the program focuses on interventions to reach the hardest-to-reach men (those over 25 years old and employed). Several studies have identified financial concerns, specifically lost wages from taking time off for surgery, as a barrier to VMMC uptake among this subgroup. An incentive-based demand creation strategy was therefore evaluated.^54^ The study found that small amounts of fixed compensation in the form of food vouchers were effective in removing structural barriers for men who had already committed to getting circumcised. The national program is currently exploring scaling-up this intervention.

Messaging often emphasizes HIV prevention as the primary benefit of VMMC, while at the same time communicating that the procedure provides only partial protection against HIV.^26^ Successful advertising positions the product or service in a way that resonates with the prospective customer rather than communicating only the most obvious features. For example, research found that anti-smoking campaigns targeting youth were most effective if the messages did not emphasize the negative long-term effects of smoking, but rather the deceptive promotional practices of cigarette manufacturers and the effects of secondhand smoke on others.^74^ Given that HIV prevention—the public health goal of VMMC—may not be the highest priority or most attractive benefit of the procedure for most males, it is important to consider positioning and messaging VMMC in ways that move beyond HIV.

Implementation experience supported by the literature shows that clients frequently cite word-of-mouth as influential in their decision to seek VMMC.^75^^,^^76^ This underscores the importance of ensuring that clients are well cared for throughout their VMMC experience. Technical excellence provided in an environment that fosters trust, affirmation, and respect increases client satisfaction and makes it more likely that early adopters will encourage their peers to opt for VMMC, in line with the theory of diffusion of innovation.^77^ Ensuring that services are not just available and accessible but also acceptable (i.e., staff are respectful and have a good bedside manner, and counseling is honest and appropriate) may help to drive demand. Maintaining customer-service standards for all facility staff providing VMMC may help generate consistently favorable client narratives about the VMMC experience. Intervention design must also take into account supply,^23^ so that the service is available and accessible to men who are persuaded by the demand generation activity.

Word-of-mouth recommendations for VMMC influence men’s decisions, thus emphasizing the need for high-quality services.

### Implementation and Coordination to Achieve Scale: Foster a Coordinated Response

Demand interventions must be scaled strategically. In order to avoid mismatches between supply and demand, partners on both sides of the equation need to use data to drive their decisions, and they must coordinate their work.^1^ Coordination minimizes confusion by ensuring that messaging is accurate, culturally appropriate, and not in contradiction with other VMMC messages likely to be encountered by the target population. Coordination between country governments and donors is also important to avoid duplication of activities and to solve infrastructure challenges. In the absence of a national VMMC operational plan, we recommend that countries establish alternative, ongoing national coordination mechanisms for all demand generation partners.

In some countries where seasonal demand is pronounced (e.g., demand is traditionally high during certain seasons of the year or campaigns during school holidays drive high uptake among adolescents), it may be necessary to change attitudes among providers as well as among the male population itself, so that resources are not underused for periods of the year, which drives up unit costs.^78^

### Measurement, Learning, and Evaluation: Evolve the Strategy Over the Course of the VMMC Program

**FIGURE 5.**
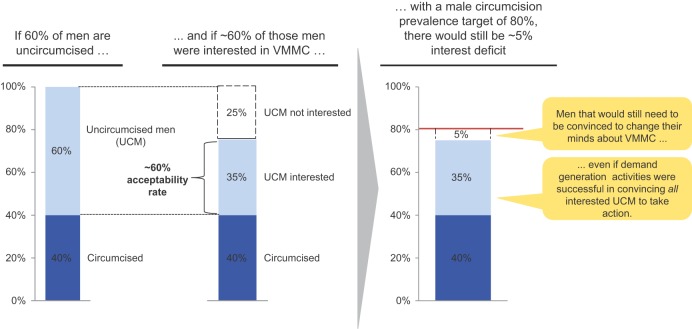
Identifying the “Interest Deficit” for Voluntary Medical Male Circumcision (VMMC)

**FIGURE 6.**
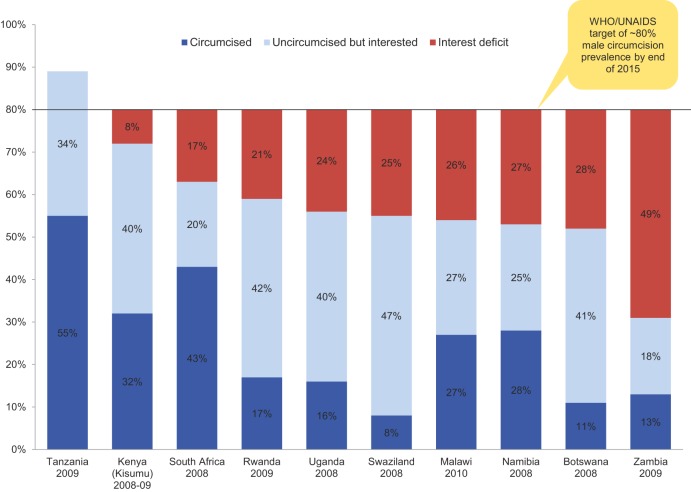
The “Interest Deficit” for Voluntary Medical Male Circumcision (VMMC) in Selected VMMC Priority Countries of Eastern and Southern Africa Data (and age range) based on country studies: Tanzania (ages 18–44),^79^ Kenya (ages 15–49),^80^ South Africa (ages 15–49),^81^ Rwanda (ages 15–59),^82^ Uganda (ages 18–80),^83^ Swaziland (ages 15–29),^84^ Malawi (ages 15 and older),^85^ Namibia (ages 15–29),^84^ Botswana (ages 15–29),^84^ Zambia (ages 15–59).^86^

There are currently no standard methods for measuring demand for VMMC, but several possible avenues could be explored. Acceptability studies can estimate the proportion of the population that is interested in VMMC and that can therefore potentially be persuaded easily to get circumcised, as well as the proportion that would need to be convinced first to change their minds about VMMC in order to reach the VMMC program target—what we refer to as the “interest deficit” (Figure 5). These subgroups will require different strategies in order to be persuaded. Our synthesis of findings from country-level studies between 2008 and 2010 indicates that the level of potential demand varies widely between countries, resulting in interest deficits ranging from 8% in Kisumu, Kenya, to 49% in Zambia (Figure 6). Ideally, market research tools should be used throughout the evolution of a VMMC program to measure interest and accurately track the changing proportions of the target population at each stage of the path toward choosing VMMC (Figure 2). Equipped with these objective data, stakeholders in each country can more accurately estimate demand for VMMC and set realistic demand generation goals.

Programs should develop and use intervention-specific indicators that can help monitor the performance of demand generation interventions well before they translate to the obvious VMMC output of circumcisions performed. For example, indicators should address such questions as: Is the intervention being implemented as designed? Is it reaching the targeted population? Could operations be more efficient? For a mass media campaign, for instance, this could include measuring exposure to and attitudes about the messages.^58^

Demand generation interventions must change over the course of a VMMC program to reflect evolving norms and preferences, with information continuously fed back into the program to identify needs for program refinement or course correction. An early adopter of VMMC may respond to different appeals than a member of the late majority. Similarly, as community knowledge and experience with VMMC increases, the information required to spur demand may change. At the outset of Kenya’s VMMC program, for example, demand generation interventions engaged leaders of the traditionally non-circumcising Luo community in Nyanza to address traditional barriers to uptake and to build a strong enabling environment. As the community-level barriers were overcome, the program turned to individual barriers by way of a large-scale, incentivized, peer-led outreach campaign.^87^

Properly evaluating the effectiveness and cost-effectiveness of interventions can help programmers design and scale-up programs to achieve the best possible results. Many of the promising practices highlighted in this paper have not yet been evaluated, and we encourage all field practitioners to formally evaluate VMMC interventions targeting specific barriers and triggers of demand, both to know how well their programs are working and to strengthen the overall evidence base informing demand generation. We also encourage a clearer definition of what constitutes sufficient evidence of impact. While demonstrating attribution, especially through RCTs, has been the gold standard,^88^ this can prove too conservative an approach in the context of VMMC. RCTs are of limited use in addressing behavior change interventions,^89^ and given the complexity of the VMMC environment, most often no single intervention is likely to be able to tackle all barriers to demand. Therefore, consideration should be given to using mixed methods to evaluate effectiveness^90^ and to judging “contribution” as a sufficient measure of effectiveness.

Studies highlight the paucity of data on the actual cost of demand generation activities.^1^^,^^47^^,^^48^^,^^91^ We recommend building rigorous yet rapid impact evaluation of demand generation interventions into program implementation processes and tracking the cost of interventions more systematically and over the course of the program. This will help programs monitor how different levels of investment affect clinic volume and cost per circumcision performed.

The dynamic environment of VMMC demand sets a high bar for measurement, learning, and evaluation strategies. Nevertheless, national governments and donors can look to strategic measurement and evaluation as an investment that is likely to pay a quick dividend. More research into the effectiveness of demand generation interventions is urgently needed. The time and money spent in this often overlooked area can replace speculative demand generation with strategic interventions for greater effect and efficiency.

## CONCLUSION

VMMC is key to achieving sustained declines in HIV incidence across eastern and southern Africa—a region with the world’s highest HIV prevalence. Averting infections, saving lives, and conserving financial and human resources that can be used to address other threats to public health is no longer a far-fetched prospect. The sooner VMMC goals are reached, the sooner countries can expect to enjoy the public health benefits this intervention offers. To get there, VMMC service delivery and demand generation must move forward in tandem. All stakeholders—governments, donors, implementing partners, and the private sector—must coordinate their work, not only to ensure demand for VMMC is balanced with supply but also to better tailor demand generation to the unique environment and circumstances of each country or region, thus ensuring successful programs.

Operations research points the way to more efficient and effective service delivery.^92^^–^^95^ It is time to apply a similar approach to demand generation, so that promising practices can be identified, tested, adapted, scaled-up, evaluated, and continually renewed. National governments, working with donors and other partners, can look to the components of demand generation described in this paper—insight development, implementation design, implementation and coordination for scale, and measurement, learning, and evaluation—as foundations for their evidence-based strategy for increasing VMMC uptake. This multidisciplinary and systematic approach to demand generation offers benefits extending beyond HIV prevention to issues across the field of health promotion.

## Notes

**Peer Reviewed**

## References

[1] Sgaier SK Reed JB Thomas A Njeuhmeli E. Achieving the HIV prevention impact of voluntary medical male circumcision: lessons and challenges for managing programs. PLoS Med. 2014;11(5):e1001641. 10.1371/journal.pmed.1001641. 24800840 PMC4011573

[2] World Health Organization (WHO); Joint United Nations Programme on HIV/AIDS. (UNAIDS). Joint strategic action framework to accelerate the scale-up of voluntary medical male circumcision for HIV prevention in Eastern and Southern Africa, 2012–2016. Geneva: UNAIDS; 2011 Available from: http://www.who.int/hiv/pub/strategic_action2012_2016/en/

[3] Njeuhmeli E Forsythe S Reed J Opuni M Bollinger L Heard N Voluntary medical male circumcision: modeling the impact and cost of expanding male circumcision for HIV prevention in eastern and southern Africa. PLoS Med. 2011;8(11):e1001132. 10.1371/journal.pmed.1001132. 22140367 PMC3226464

[4] World Health Organization (WHO). Draft summary from WHO informal consultation on tetanus and voluntary medical male circumcision, 9–10 March 2015. Draft version 5.1. Geneva: WHO; 2015.

[5] Clearinghouse on Male Circumcision for HIV Prevention [Internet]. New York: AIDS Vaccine Advocacy Coalition; c2015. Campaign materials from ‘Creating Demand for VMMC’ website [cited 2015 Apr 2] Available from: https://www.malecircumcision.org/demand-creation/campaign-materials

[6] Galdas PM Cheater F Marshall P. Men and health help-seeking behaviour: literature review. J Adv Nurs. 2005;49(6):616 623. 10.1111/j.1365-2648.2004.03331.x. 15737222

[7] Clearinghouse on Male Circumcision for HIV Prevention [Internet]. New York: AIDS Vaccine Advocacy Coalition; c2015. Eastern and Southern Africa Regional Meeting on Demand Creation for Voluntary Medical Male Circumcision, Lusaka, Zambia, April 3–5, 2013 [cited 2015 Jan 18] Available from: http://www.malecircumcision.org/communication/Zambia_Regional_Meeting_April2013.html

[8] Prochaska JO DiClemente CC Norcross JC. In search of how people change: applications to addictive behaviors. Am Psychol. 1992;47(9):1102–1114. 10.1037/0003-066X.47.9.1102. 1329589

[9] Schwarzer R Modeling health behavior change: How to predict and modify the adoption and maintenance of health behaviors. Appl Psychol Int Rev. 2008;57:1–29. 10.1111/j.1464-0597.2007.00325.x

[10] Sutton S Stage theories of health behaviour. Conner M Norman P, Predicting health behaviour: research and practice with social cognition models. 2nd ed Maidenhead (UK): Open University Press; 2005.

[11] Montaño DE Kasprzyk D Hamilton DT Tshimanga M Gorn G. Evidence-based identification of key beliefs explaining adult male circumcision motivation in Zimbabwe: targets for behavior change messaging. AIDS Behav. 2014;18(5):885–904. 10.1007/s10461-013-0686-7. 24443147 PMC3984408

[12] Macintyre K Andrinopoulos K Moses N Bornstein M Ochieng A Peacock E Attitudes, perceptions and potential uptake of male circumcision among older men in Turkana County, Kenya using qualitative methods. PLoS One. 2014;9(5):e83998. 10.1371/journal.pone.0083998. 24802112 PMC4011674

[13] Hatzold K Mavhu W Jasi P Chatora K Cowan FM Taruberekera N Barriers and motivators to voluntary medical male circumcision uptake among different age groups of men in Zimbabwe: results from a mixed methods study. PLoS One. 2014;9(5):e85051. 10.1371/journal.pone.0085051. 24802746 PMC4011705

[14] Ashengo TA Hatzold K Mahler H Rock A Kanagat N Magalona S Voluntary medical male circumcision (VMMC) in Tanzania and Zimbabwe: service delivery intensity and modality and their influence on the age of clients. PLoS One. 2014;9(5):e83642. 10.1371/journal.pone.0083642. 24801882 PMC4011872

[15] Djimeu Wouabe E Scoping report on interventions for increasing the demand for voluntary medical male circumcision. Washington (DC): International Initiative for Impact Evaluation (3IE); 2013 Available from: www.3ieimpact.org/media/filer/2013/03/22/white_paper__vmmc.pdf

[16] Clearinghouse on Male Circumcision for HIV Prevention [Internet]. New York: AIDS Vaccine Advocacy Coalition; c2015. Promising practices [cited 2015 Jan 18] Available from: http://www.malecircumcision.org/communication/promising_practices.html

[17] Westercamp N Bailey RC. Acceptability of male circumcision for prevention of HIV/AIDS in sub-Saharan Africa: a review. AIDS Behav. 2007;11(3):341–355. 10.1007/s10461-006-9169-4. 17053855 PMC1847541

[18] Westercamp M Agot KE Ndinya-Achola J Bailey RC Circumcision preference among women and uncircumcised men prior to scale-up of male circumcision for HIV prevention in Kisumu, Kenya. AIDS Care. 2012;24(2):157–166. 10.1080/09540121.2011.597944 Medline 21854351 PMC3682798

[19] Simbayi L Peltzer K Onoya D Prevalence and acceptability of male circumcision in South Africa. Presented at: 10th AIDS Impact Conference; 2011 Sep 12-15; Santa Fe, NM. Available from: www.hsrc.ac.za/en/research-outputs/ktree-doc/9974 10.4314/ajtcam.v11i4.19 PMC4202407 25392591

[20] Central Statistical Office (CSO) [Zambia]; Ministry of Health (MOH) [Zambia]; National HIV/AIDS/STI/TB Council [Zambia]; University of Zambia; MEASURE Evaluation. Zambia sexual behaviour survey 2009. Lusaka: CSO; 2010 Available from: http://www.cpc.unc.edu/measure/publications/tr-10-73

[21] Wambura M Mwanga J Mosha J Mshana G Mosha F Changaluc J Situation analysis for male circumcision in Tanzania. Dar es-Salaam (Tanzania): National Institute for Medical Research (Mwanza) and Ministry of Health and Social Welfare; 2009.

[22] Population Services International (PSI), Research and Metrics. Zimbabwe (2013): voluntary medical male circumcision TRaC study among men and women aged 15-49 years in Zimbabwe. Washington (DC): PSI; 2013.

[23] Price JE Phiri L Mulenga D Hewett PC Topp SM Shiliya N Behavior change pathways to voluntary medical male circumcision: narrative interviews with circumcision clients in Zambia. PLoS One. 2014;9(11):e111602. 10.1371/journal.pone.0111602. 25375790 PMC4222873

[24] World Bank. World development report 2015: mind, society, and behavior. Washington (DC): World Bank; 2014 10.1596/978-1-4648-0342-0

[25] Malawi News Agency. Ngoni chiefs rise to the challenge, accept male circumcision in Malawi AIDS fight. 2012.

[26] Clearinghouse on Male Circumcision for HIV Prevention [Internet]. New York: AIDS Vaccine Advocacy Coalition; c2015. Uganda: STAR-E: SMC campaign in traditionally circumcising communities “Be the Pride of Your Tribe” [cited 2015 Apr 24].

[27] Clearinghouse on Male Circumcision for HIV Prevention [Internet]. New York: AIDS Vaccine Advocacy Coalition; c2015. Research led message development for “SMART” campaign and management of social mobilization (PSI Zimbabwe) [cited 2015 Jan 18].

[28] Clearinghouse on Male Circumcision for HIV Prevention [Internet]. New York: AIDS Vaccine Advocacy Coalition; c2015. South Africa: CareWorks [cited 2015 Jan 18].

[29] Clearinghouse on Male Circumcision for HIV Prevention [Internet]. New York: AIDS Vaccine Advocacy Coalition; c2015. Lilongwe district VMMC scale-up (I-TECH, Malawi) [cited 2015 Jan 18].

[30] Clearinghouse on Male Circumcision for HIV Prevention [Internet]. New York: AIDS Vaccine Advocacy Coalition; c2015. Kenya: IRDO, creating a demand creation toolkit [cited 2015 Apr 24].

[31] Clearinghouse on Male Circumcision for HIV Prevention [Internet]. New York: AIDS Vaccine Advocacy Coalition; c2015. Zambia: SFH community mobilisation, mid media campaigns and national communications materials [cited 2015 Apr 24].

[32] Internews. Women play increasing role in male circumcision programmes. Internews [Internet]. 2012 Jul 22 [cited 2015 Jan 18] Available from: http://www.internews.org/our-stories/news/women-play-increasing-role-male-circumcision-programmes

[33] Clearinghouse on Male Circumcision for HIV Prevention [Internet]. New York: AIDS Vaccine Advocacy Coalition; c2015. Training journalists to report accurately and fairly on VMMC (Internews, Kenya) [cited 2015 Jan 18].

[34] Clearinghouse on Male Circumcision for HIV Prevention [Internet]. New York: AIDS Vaccine Advocacy Coalition; c2015. Zambia: communications support for health: mass and local media and civil society organisations [cited 2015 Jan 18].

[35] Clearinghouse on Male Circumcision for HIV Prevention [Internet]. New York: AIDS Vaccine Advocacy Coalition; c2015. Tanzania JHU–CCP: printed materials and radio spots [cited 2015 Jan 18].

[36] Clearinghouse on Male Circumcision for HIV Prevention [Internet]. New York: AIDS Vaccine Advocacy Coalition; c2015. Uganda: Makerere University Walter Reed Project (MUWRP): using cross-country learning to develop new technologies to create SMC demand and meet SMC needs [cited 2015 Jan 18].

[37] Clearinghouse on Male Circumcision for HIV Prevention [Internet]. New York: AIDS Vaccine Advocacy Coalition; c2015. Tanzania: Jhpiego Tanzania: community mobilization for “older men” and use of SMS and GIS technology [cited 2015 Jan 18].

[38] RTI International; Population Services International (PSI); US Centers for Disease Control and Prevention (CDC). Voluntary medical male circumcision (VMMC) demand creation toolkit. Washington (DC): US President’s Emergency Plan for AIDS Relief (PEPFAR); [200?]. Available from: http://www.thehealthcompass.org/sites/default/files/strengthening_tools/VMMC_Demand%20Creation%20ToolkitFINAL%20PEPFAR%20APPROVED.pdf

[39] Lissouba P Taljaard D Rech D Adult male circumcision as an intervention against HIV: An operational study of uptake in a South African community (ANRS 12126). BMC Infect Dis. 2011;11(1):253. 10.1186/1471-2334-11-253. 21943076 PMC3192707

[40] Clearinghouse on Male Circumcision for HIV Prevention [Internet]. New York: AIDS Vaccine Advocacy Coalition; c2015. Uganda: Rakai “Stylish Man” Campaign: combining traditional and new approaches to demand creation for safe male circumcision (SMC) [cited 2015 Apr 24].

[41] Clearinghouse on Male Circumcision for HIV Prevention [Internet]. New York: AIDS Vaccine Advocacy Coalition; c2015. South Africa: the Centre for HIV/AIDS Prevention Studies (CHAPS) [cited 2015 Apr 24].

[42] van den Putte B Yzer M Southwell BG de Bruijn GJ Willemsen MC. Interpersonal communication as an indirect pathway for the effect of antismoking media content on smoking cessation. J Health Commun. 2011;16(5):470–485. 10.1080/10810730.2010.546487. 21337250

[43] Frank LB Chatterjee JS Chaudhuri ST Lapsansky C Bhanot A Murphy ST. Conversation and compliance: role of interpersonal discussion and social norms in public communication campaigns.J Health Commun. 2012;17(9):1050–1067. 10.1080/10810730.2012.665426. 22808934

[44] Ensor T Cooper S. Overcoming barriers to health service access: influencing the demand side. Health Policy Plan. 2004;19(2):69–79. 10.1093/heapol/czh009. 14982885

[45] Ministry of Public Health & Sanitation [Kenya]. Kenya national strategy for voluntary medical male circumcision. Nairobi: National AIDS & STD Control Programme; 2009.

[46] Ministry of Health [Zambia]. Operational plan for the scale-up of voluntary medical male circumcision in Zambia 2012–2015. Lusaka: Ministry of Health; 2012.

[47] Bertrand JT Njeuhmeli E Forsythe S Mattison SK Mahler H Hankins CA. Voluntary medical male circumcision: a qualitative study exploring the challenges of costing demand creation in eastern and southern Africa. PLoS One. 2011;6(11):e27562. 10.1371/journal.pone.0027562. 22140450 PMC3226625

[48] Chandrashekar S Guinness L Kumaranayake L Reddy B Govindraj Y Vickerman P The effects of scale on the costs of targeted HIV prevention interventions among female and male sex workers, men who have sex with men and transgenders in India. Sex Transm Infect. 2010;86 Suppl 1:i89–i94. 10.1136/sti.2009.038547. 20167740 PMC3252618

[49] Vassal A Chandrashekar S Pickles M Towards a comprehensive approach to HIV prevention for female sex workers: the cost effectiveness of community mobilization and empowerment interventions for female sex workers in Southern India. PLoS Med. Under review. 10.1371/journal.pone.0110562 PMC4204894 25333501

[50] Bollinger L Adesina A Forsythe S Godbole R Reuben E Njeuhmeli E. Cost drivers for voluntary medical male circumcision using primary source data from sub-Saharan Africa. PLoS One. 2014;9(5):e84701. 10.1371/journal.pone.0084701. 24802593 PMC4011577

[51] Menon V Gold E Godbole R Castor D Mahler H Forsythe S Costs and impacts of scaling up voluntary medical male circumcision in Tanzania. PLoS One. 2014;9(5):e83925. 10.1371/journal.pone.0083925. 24802022 PMC4011575

[52] Clearinghouse on Male Circumcision for HIV Prevention [Internet]. New York: AIDS Vaccine Advocacy Coalition; c2015. Kenya: NASCOP & MCC, national and provincial coordination in Kenya [cited 2015 Jan 18].

[53] Kaufman ZA DeCelles J Bhauti K Weiss HA Hatzold K Chaibva C A sport-based intervention to increase uptake of voluntary medical male circumcision among adult male football players: results from a cluster-randomised trial in Bulawayo, Zimbabwe. Poster presented at: 20th International AIDS Conference, Melbourne, Australia; 2014 Jul 20-25 Available from: http://pag.aids2014.org/Abstracts.aspx?AID = 5834

[54] Thirumurthy H Masters SH Rao S Bronson MA Lanham M Omanga E Effect of providing conditional economic compensation on uptake of voluntary medical male circumcision in Kenya: a randomized clinical trial. JAMA. 2014;312(7):703–711. 10.1001/jama.2014.9087. 25042290 PMC4268484

[55] International Initiative for Impact Evaluation (3ie) [Internet]. New Delhi (India): 3ie; c2012. Thematic window 3 award winners [cited 2015 Jan 18] Available from: http://www.3ieimpact.org/en/funding/thematic-window/thematic-window-3-voluntary-male-medical-circumcision/thematic-window-3-award-winners/

[56] Rosenstock IM The health belief model and preventive health behavior. Health Educ Behav. 1974;2:354–386.

[57] de Vries H Dijkstra M Kuhlman P Self-efficacy: the third factor besides attitude and subjective norm as a predictor of behavioural intentions. Health Educ Res. 1988;3(3):273–282. 10.1093/her/3.3.273

[58] McLeroy KR Bibeau D Steckler A Glanz K. An ecological perspective on health promotion programs.Health Educ Behav. 1988;15(4):351–377. 10.1177/109019818801500401. 3068205

[59] Stokols D. Translating social ecological theory into guidelines for community health promotion. Am J Health Promot. 1996;10(4):282–298. 10.4278/0890-1171-10.4.282. 10159709

[60] Richard L Potvin L Kishchuk N Prlic H Green LW. Assessment of the integration of the ecological approach in health promotion programs.Am J Health Promot. 1996;10(4):318–328. 10.4278/0890-1171-10.4.318. 10159711

[61] Iyengar S The art of choosing. New York: Twelve; 2010.

[62] Camerer CF Loewenstein G Behavioral economics: past, present, future. In: Camerer CF, Loewenstein G, Rabin M, editors. Advances in behavioral economics. Princeton (NJ): Princeton University Press; 2004.

[63] Banerjee AV Duflo E Glennerster R Kothari D. Improving immunisation coverage in rural India: clustered randomised controlled evaluation of immunisation campaigns with and without incentives. BMJ. 2010;340:c2220. 10.1136/bmj.c2220. 20478960 PMC2871989

[64] Creative for Good [Internet]. Geneva: World Economic Forum. Jimerson A. Talking babies for breastfeeding (Vietnam); [cited 2015 Apr 2]. Available from http://www.weforum.org/best-practices/creative-good/talking-babies-breastfeeding-vietnam

[65] Hawkins DI Mothersbaugh D Consumer behavior: building marketing strategy. 11th ed. New York: McGraw-Hill/Irwin; 2010.

[66] McDonald M Marketing plans: how to prepare them, how to use them. 6th ed. Oxford: Butterworth-Heinemann; 2007.

[67] Janiszewska K Insch A The strategic importance of brand positioning in the place brand concept: elements, structure and application capabilities. J Int Stud. 2012;5(1):9–19. 10.14254/2071-8330.2012/5-1/2

[68] Malhotra N Peterson M Kleiser SB Marketing research: a state-of-the-art review and directions for the twenty-first century. J Acad Market Science. 1999;27(2):160–83. 10.1177/0092070399272004

[69] Frew PM Painter JE Hixson B Kulb C Moore K del Rio C Factors mediating seasonal and influenza A (H1N1) vaccine acceptance among ethnically diverse populations in the urban south. Vaccine. 2012;30(28):4200–4208. 10.1016/j.vaccine.2012.04.053. 22537991 PMC3522428

[70] Geeroms N Towards a better understanding of motivational consumer behavior: cross-validation, construct validation and application of a psychological taxonomy of consumer motives. Ghent (Belgium): Ghent University; 2007.

[71] Nieuwoudt S Frade S Rech D Taljaard D Uncovering the dirt on demand creation for medical circumcision. Johannesburg (South Africa): Centre for HIV and AIDS Prevention Study (CHAPS); 2012.

[72] Ananya [Internet]. New Delhi: BBC Media Action. Mobile Kunji [cited 2015 Jan 18] Available from: http://www.rethink1000days.org/programme-outputs/mobile-kunji/

[73] Bill & Melinda Gates Foundation. Managing HIV prevention from the ground up: Avahan’s experience with peer led outreach at scale in India. New Delhi: The Foundation; 2009 Available from: https://docs.gatesfoundation.org/Documents/peer-led-outreach.pdf

[74] Pechmann C Reibling ET. Anti-smoking advertising campaigns targeting youth: case studies from USA and Canada. Tob Control. 2000;9 Suppl 2:II18–31. 10.1136/tc.9.suppl_2.ii18. 10841588 PMC1766281

[75] Nielsen Newswire [Internet]. New York: Nielsen; c2015. Why word-of-mouth is loudest in Africa. 2013 Apr 3 [cited 2015 Jan 18] Available from: http://www.nielsen.com/us/en/newswire/2013/why-word-of-mouth-is-loudest-in-africa.html

[76] Galukande M Sekavuga DB Duffy K Wooding N Rackara S Nakaggwa F Mass safe male circumcision: early lessons from a Ugandan urban site - a case study. Pan Afr Med J. 2012;13:88. 23396906 PMC3567401

[77] Rogers E Diffusion of Innovations. 5th ed. New York: Free Press; 2003.

[78] Gold E Mahler H Boyee D Overcoming seasonality in scaling up voluntary medical male circumcision: a case study from Tanzania. Arlington (VA): Strengthening High Impact Interventions for an AIDS-free Generation (AIDSFree) Project; 2015 Available from: http://www.healthcommcapacity.org/wp-content/uploads/2015/02/Tanzania-Seasonality.pdf

[79] Wambura M Mwanga J Mosha J Mshana G Mosha F Changalucha J Situation analysis for male circumcision in Tanzania: final report. Mwanza (Tanzania): National Institute for Medical Research [Tanzania]; 2009. Co-published by Ministry of Health and Social Welfare [Tanzania].

[80] Westercamp M Agot KE Ndinya-Achola J Bailey RC. Circumcision preference among women and uncircumcised men prior to scale-up of male circumcision for HIV prevention in Kisumu, Kenya. AIDS Care. 2012;24(2):157–166. 10.1080/09540121.2011.597944. 21854351 PMC3682798

[81] Peltzer K Onoya D Makonko E Simbayi L. Prevalence and acceptability of male circumcision in South Africa. Afr J Tradit Complement Altern Med. 2014;11(4):126–130. 25392591 10.4314/ajtcam.v11i4.19 PMC4202407

[82] Gasasira RA Sarker M Tsague L Nsanzimana S Gwiza A Mbabazi J Determinants of circumcision and willingness to be circumcised by Rwandan men, 2010. BMC Public Health. 2012;12:134. 10.1186/1471-2458-12-134. 22340083 PMC3299639

[83] Albert LM Akol A L'Engle K Tolley EE Ramirez CB Opio A Acceptability of male circumcision for prevention of HIV infection among men and women in Uganda. AIDS Care. 2011;23(12):1578–1585. 10.1080/09540121.2011.579939. 21732902

[84] Andersson N Cockcroft A. Male circumcision, attitudes to HIV prevention and HIV status: a cross-sectional study in Botswana, Namibia and Swaziland. AIDS Care. 2012;24(3):301–309. 10.1080/09540121.2011.608793. 21933035 PMC3379742

[85] Bengo JM Chalulu K Chinkhumba J Kazembe L Maleta KM Masiye F Situation analysis of male circumcision in Malawi: a report prepared by the College of Medicine. Blantyre (Malawi): University of Malawi College of Medicine; 2010 Available from: http://www.researchgate.net/profile/Joseph_Mfutso-Bengo/publication/262062700_Final_report_for_the_Situation_analysis_of_Male_circumcision/links/0c9605368c21ca2f81000000

[86] Central Statistical Office (CSO) [Zambia]; Ministry of Health [Zambia]; University of Zambia; MEASURE Evaluation. Zambia sexual behaviour survey 2009. Lusaka (Zambia): CSO; 2009. Co-published by MEASURE Evaluation Available from: http://www.cpc.unc.edu/measure/publications/tr-10-73

[87] Mwandi Z Murphy A Reed J Chesang K Njeuhmeli E Agot K Voluntary medical male circumcision: translating research into the rapid expansion of services in Kenya, 2008-2011. PLoS Med. 2011;8(11):e1001130. 10.1371/journal.pmed.1001130. 22140365 PMC3226459

[88] Padian NS McCoy SI Balkus JE Wasserheit JN. Weighing the gold in the gold standard: challenges in HIV prevention research. AIDS. 2010;24(5):621–635. 10.1097/QAD.0b013e328337798a. 20179575 PMC3695696

[89] Over M Achieving an AIDS transition: preventing infections to sustain treatment. Washington (DC): Center for Global Development; 2001 Available from: http://www.cgdev.org/publication/9781933286389-achieving-aids-transition-preventing-infections-sustain-treatment

[90] Greene JC Caracelli VJ Graham WF Toward a conceptual framework for mixed-method evaluation designs. Educ Eval Policy Anal. 1989;11(3):255–274. 10.3102/01623737011003255

[91] Njeuhmeli E Kripke K Hatzold K Reed J Edgil D Jaramillo J Cost analysis of integrating the PrePex medical device into a voluntary medical male circumcision program in Zimbabwe. PLoS One. 2014;9(5):e82533. 10.3102/01623737011003255. 24801515 PMC4011574

[92] Rech D Bertrand JT Thomas N Farrell M Reed J Frade S Surgical efficiencies and quality in the performance of voluntary medical male circumcision (VMMC) procedures in Kenya, South Africa, Tanzania, and Zimbabwe. PLoS One. 2014;9(5):e84271. 10.1371/journal.pone.0084271. 24802412 PMC4011873

[93] Bertrand JT Rech D Omondi Aduda D Frade S Loolpapit M Machaku MD Systematic monitoring of voluntary medical male circumcision scale-up: adoption of efficiency elements in Kenya, South Africa, Tanzania, and Zimbabwe. PLoS One. 2014;9(5):e82518. 10.1371/journal.pone.0082518. 24801374 PMC4011576

[94] Rech D Spyrelis A Frade S Perry L Farrell M Fertziger R Implications of the fast-evolving scale-up of adult voluntary medical male circumcision for quality of services in South Africa. PLoS One. 2014;9(5):e80577. 10.1371/journal.pone.0080577. 24801209 PMC4011681

[95] Jennings L Bertrand J Rech D Harvey SA Hatzold K Samkange CA Quality of voluntary medical male circumcision services during scale-up: a comparative process evaluation in Kenya, South Africa, Tanzania and Zimbabwe. PLoS One. 2014;9(5):e79524. 10.1371/journal.pone.0079524. 24801073 PMC4011679

